# Amelioration of experimental autoimmune encephalomyelitis by clozapine is not associated with defective CD4 T cell responses

**DOI:** 10.1186/s12974-017-0842-5

**Published:** 2017-03-29

**Authors:** Pirooz Zareie, Bronwen Connor, Anne Camille La Flamme

**Affiliations:** 1 Centre for Biodiscovery, School of Biological Sciences, Victoria University of Wellington, P.O. Box 600, Wellington, 6140 New Zealand; 2 Department of Pharmacology and Clinical Pharmacology, Centre for Brain Research, University of Auckland, Auckland, New Zealand; 3 The Malaghan Institute of Medical Research, Wellington, New Zealand

**Keywords:** EAE, MS, Atypical antipsychotics, Neuroinflammation

## Abstract

Atypical antipsychotic agents, such as clozapine, are used for treating psychosis and depression and have recently been found to modulate neuroinflammation. We have shown previously that treatment of mice with the atypical antipsychotic agents, clozapine or risperidone, attenuates disease severity in experimental autoimmune encephalomyelitis (EAE); however, the mechanism by which they are protective is unknown. In this study, we investigated the effects of clozapine on CD4^+^ T cell responses and found that clozapine did not significantly affect the expansion of myelin-specific T cells, their differentiation into pathogenic subsets, or their encephalitogenic capacity to induce EAE. Interestingly, although clozapine enhanced differentiation of regulatory T (Treg) cells, in vivo neutralization of Tregs indicated that Tregs were not responsible for the protective effects of clozapine during the induction and effector phase of EAE. Taken together, our studies indicate that clozapine does not mediate its protective effects by directly altering CD4 T cells.

**Electronic supplementary material:**

The online version of this article (doi:10.1186/s12974-017-0842-5) contains supplementary material, which is available to authorized users.

## Introduction

Multiple sclerosis (MS) is a debilitating disease of the central nervous system (CNS) that is mediated by inflammatory demyelination of the myelin sheaths that surround neuronal axons [1]. Immune-mediated demyelination impairs nerve conduction and causes the clinical symptoms of MS, which are diverse and include loss of coordination, visual disturbances, fatigue, and paralysis; the degree of which is assessed using the Expanded Disability Status Scale [2]. Although there are now a number of therapeutic options for relapsing-remitting MS patients, they are not similarly effective in all patients and only one has shown potential in the primary progressive forms of MS. This is not surprising given that blood-brain barrier (BBB) disruption is minimal in the progressive forms [3] and current therapeutic strategies are protein - based such as glatiramer acetate, interferon-β, and natalizumab, which have limited capacity to cross the intact BBB [4]. In addition, while ocrelizumab has shown limited potential in the treatment of primary progressive MS [5], its efficacy in patients may partly be determined by the severity of BBB disruption. Therefore, effective therapeutic options are urgently needed.

MS is considered a neuroinflammatory disease and shares some pathophysiological similarities with several neuropsychiatric disorders. For example, it is becoming increasingly evident that neuropsychiatric disorders such as schizophrenia and major depressive disorders are associated with inflammation in the CNS and characterized by chronic microglial activation [6]. These diseases are associated with elevated inflammatory markers and, in particular, the expression of inflammatory cytokines interleukin (IL-) 1β, IL-6, and tumor necrosis factor-α, which were found in post-mortem samples from suicide victims with major depression [7] and schizophrenia [8]. Clinical treatments for psychiatric disease like fluoxetine, risperidone, quetiapine, and clozapine have recently been acknowledged for their immunomodulatory effects in various models of inflammation [9–12] and show promise as immune-modulating agents. Abnormal serum cytokine levels in schizophrenic patients are normalized by treatment with atypical antipsychotics in some studies [13], suggesting an immune - altering effect.

Recently, we have shown that the atypical antipsychotic agents risperidone and clozapine are effective at reducing disease in experimental autoimmune encephalomyelitis (EAE) [10], demonstrating that atypical antipsychotic agents are potential treatments for MS. While these agents show promise as therapeutics in MS and other neuroinflammatory disorders, the mechanism by which they reduce disease and alter inflammation is not completely understood. Given that EAE is a disease that is driven by autoreactive Th1 and Th17 cells and that defective development of these subsets greatly alters the susceptibility of mice to EAE, this study aimed to investigate if clozapine were able to reduce disease by impairing CD4 T cell-mediated induction of EAE.

## Materials and methods

### Mice

All mice used were female and aged between 8 and 12 weeks. C57BL6/J were purchased from the Biomedical Research Unit of the Malaghan Institute of Medical Research (Wellington, NZ) and housed in the Victoria University of Wellington animal facility. 2D2 TCR^MOG35-55^ (CD45.2) mice expressing the T cell receptor (TCR) specific for myelin oligodendrocyte glycoprotein (MOG) 35-55 and B6-SJ^ptprca^ (CD45.1) mice were bred at the Victoria University of Wellington animal facility. Food and water were available *ad libitum*.

### Induction of EAE and clozapine treatment

#### Active EAE

EAE was induced by subcutaneous (s.c) immunization in the rear flanks of mice with MOG_35-55_ (50 μg/mouse; Genscript, Piscataway, NJ, USA) emulsified in complete Freund’s adjuvant (500 μg/mouse *Mycobacterium tuberculosis*) followed by intraperitoneal (i.p) injection of 200 ng/mouse pertussis toxin on days 0 and 2. Mice were weighed and scored daily for signs of disease by a non-blinded investigator, using the following disease rating scale: 0, normal; 1, partial tail paralysis; 2, full tail paralysis; 3, full paralysis of one hind limb; 4, full paralysis of both hind limbs; and 5, moribund. Clozapine was kindly supplied by Douglas Pharmaceuticals Ltd. (Auckland, New Zealand) and added to drinking water 1 day prior to EAE induction at a concentration calculated to achieve a daily dose of 60 mg/kg. For regulatory T (Treg) neutralization, mice were injected with 200 μg of anti-CD25 monoclonal antibody (PC61; BioXCell, West Lebanon, NH, USA) or rat IgG (Sigma-Aldrich, St. Louis, MO, USA) i.p 3 days prior to EAE induction and maintained with repeat injections at 7 and 14 days post immunization (d.p.i).

#### Adoptively transferred EAE

Donor C57BL6/J mice were immunized for EAE as described above but without the pertussis toxin. Spleens and draining lymph nodes were harvested at 12 d.p.i, and single-cell suspensions were made by passage through a 70-μm cell strainer (BD Biosciences, Franklin Lakes, NJ). Red blood cells were lysed using Red Blood Cell Lysis Buffer Hybri-Max (Sigma-Aldrich, St. Louis, MO). Splenocytes were washed and re-suspended in culture medium containing Dulbecco’s modified Eagle medium (DMEM), 100 U/mL penicillin, 100 μg/mL streptomycin, 10 mM HEPES, 2 mM l-glutamine, 50 μM 2-mercaptoethanol, non-essential amino acids and 10% fetal calf serum (all from Life Technologies, Carlsbad, CA, USA). Donor cells were cultured in tissue culture flasks at 1 × 10^7^ cells/mL with IL-12p70 (20 ng/mL; Peprotech, Rocky Hill, NJ, USA), XMG1.2 (10 μg/mL; BioXCell, USA), MOG_35-55_ (50 μg/mL), and either vehicle or clozapine (20 μM) at 37 °C and 5% CO_2_. After 96 h of stimulation, non-adherent donor cells were harvested and injected into recipient naïve C57BL/6J mice (2 × 10^7^ cells/mouse) followed by pertussis toxin (200 ng/mouse) on days 0 and 2. Mice were monitored daily for disease as described above.

### In vivo proliferation assay

Splenocytes and lymph node cells were isolated from 2D2 TCR^MOG35-55^ mice as described and labelled with CellTrace CFSE Cell Proliferation Kit (Life Technologies) according to the manufacturer’s protocol. CFSE-labelled 2D2 TCR^MOG35-55^ cells (2 × 10^7^) were injected i.p into B6-SJ^ptprca^ 1 day prior to EAE induction. At day 0, mice were immunized to induce EAE and euthanized 5 days post immunization. Proliferation of CD45.2^+^CD4^+^ cells were assessed in peripheral blood (cardiac puncture), draining lymph nodes (inguinal and mesenteric), and spleen by flow cytometry.

### In vitro assays

Splenocytes, isolated as described above, were seeded in flat bottom 96-well plates (BD Biosciences) at 1 × 10^6^ cells/well. Splenocytes were stimulated with MOG_35-55_ (50 μg/mL) (Genscript) for 72 h before supernatant was collected for cytokine detection.

### T cell differentiation

Splenocytes were plated at 1 × 10^6^ cells/well in flat bottom 96-well plates (BD Biosciences). Th1 cells were induced by addition of IL-12p70 (20 ng/mL) and 11B11 (10 μg/mL; a gift from the Malaghan Institute of Medical Research). Th17 cells were induced by addition of transforming growth factor (TGF-) β1 (5 ng/mL; eBioscience, San Diego, CA), IL-6 (20 ng/mL; BD Biosciences), and XMG1.2 (10 μg/mL; BioXCell). Tregs were induced by addition of TGF-β1 (5 ng/mL), MP5-20F3 (10 μg/mL), XMG1.2 (10 μg/mL), and 11B11 (10 μg/mL). Clozapine or vehicle (AcOH) was added at indicated final concentrations. Cells were incubated for 72 h at 37 °C and 5% CO_2_ after which supernatant was collected and T cells were stimulated with 50 ng/mL phorbol 12-myristate 13-acetate (PMA), 500 ng/mL ionomycin (Sigma-Aldrich) and Golgi Stop (BD Biosciences) used according to the manufacturer’s recommendations for a further 5 h before analysis of T cell subsets by intracellular flow cytometry.

### Flow cytometry

For the detection of immune cells, cytokines, and transcription factors, the following antibodies were used: CD4 (GK1.5; Biolegend, San Diego, CA, USA), CD45 (30-F11; Biolegend), CD25 (PC61; Biolegend), CD8 (53-6.7; Biolegend), CD11b (M1/70; Biolegend), CD3 (17A2; Biolegend), IFN-γ (XMG1.2; BD Biosciences), T-bet (04-46; BD Biosciences), RORγT (Q31-378; BD Biosciences), IL-17A (TC11-18H10; BD Biosciences), Foxp3 (150D; Biolegend), CD45.1 (A20; BD Biosciences), CD45.2 (104; BD Biosciences), goat antirabbit FITC (BD Biosciences), and rabbit antimouse dopamine D1 and D2 receptor (both from Merck, Darmstadt, Germany). For cell surface staining, cells were incubated with Fc Block (2.4G2; BD Biosciences) for 15 min prior to staining with fluorescently labelled antibodies for 20 min on ice. For intracellular staining, cells were fixed, permeabilized, and stained using Transcription Factor Buffer Set (BD Biosciences) according to the manufacturer’s protocol. Flow cytometry was performed on a BD FACS Canto II (BD Biosciences) and analyzed using Flowjo software (Treestar Inc., Ashland, OR, USA).

### Cytokine measurement

Cytokines in the supernatant were measured by LEGENDplex Mouse Inflammation Panel multi-analyte flow assay kit (Biolegend) or sandwich ELISA (BD Biosciences) according to the manufacturer’s recommendations.

### Statistics

Data were graphed and analyzed by one-way ANOVA, two-way ANOVA, Student’s *t* test, and Mann-Whitney test as indicated in each figure using GraphPad Prism software (GraphPad, La Jolla, CA, USA). Dunnett’s and Sidak’s post hoc multiple comparison test was performed to determine significant differences between test groups. *P* values of <0.05 were considered significant.

## Results

### Clozapine treatment delayed onset and reduced severity of EAE

**Fig. 1:**
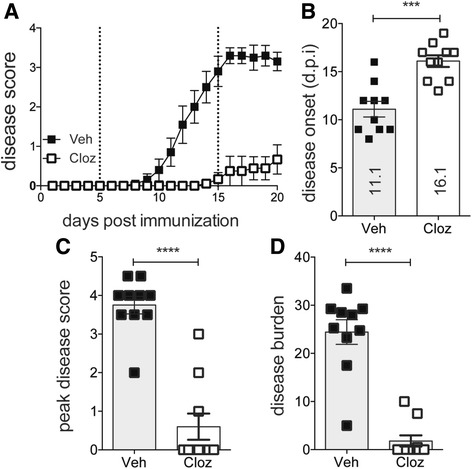
Clozapine delayed the onset and reduced disease severity of EAE but did not alter antigen-specific T cell expansion. Mice were treated with clozapine or vehicle 1 day prior to EAE induction and scored daily (0: normal to 5: moribund). **a** Disease course of mice. **b** Disease onset. **c** Peak disease score. **d** Disease burden assessed by area under the curve analysis. Shown are the means and SEM of individual mice from two experiments combined (*n* = 10 mice/group). ****p < 0.0001 by Mann-Whitney test

It has recently been shown that atypical antipsychotics such as risperidone, clozapine, and quetiapine are able to modulate the immune response and reduce disease effectively during EAE [10, 11]; however, the mechanism by which they mediate this effect is not yet fully understood. To better understand this mechanism, we used a dose of 60 mg/kg/day clozapine starting the day before immunization to effectively reduce the disease course of EAE (Fig. 1a), and this dose has also been shown recently to work therapeutically when administered at 12 or 20 days post immunization (d.p.i) [14]. Consistent with previous findings, vehicle-treated mice developed clinical symptoms of EAE with a mean onset of disease at 11 d.p.i whereas clozapine-treated mice experienced a delayed disease onset with a mean of 16 d.p.i (Fig. 1b). Mice that received clozapine had reduced peak disease score (Fig. 1c), and this contributed to an overall reduction in disease burden (Fig. 1d). These results demonstrate that clozapine is effective at reducing and delaying EAE disease.

### Treatment with clozapine did not alter MOG-specific T cell expansion upon EAE induction

**Fig. 2:**
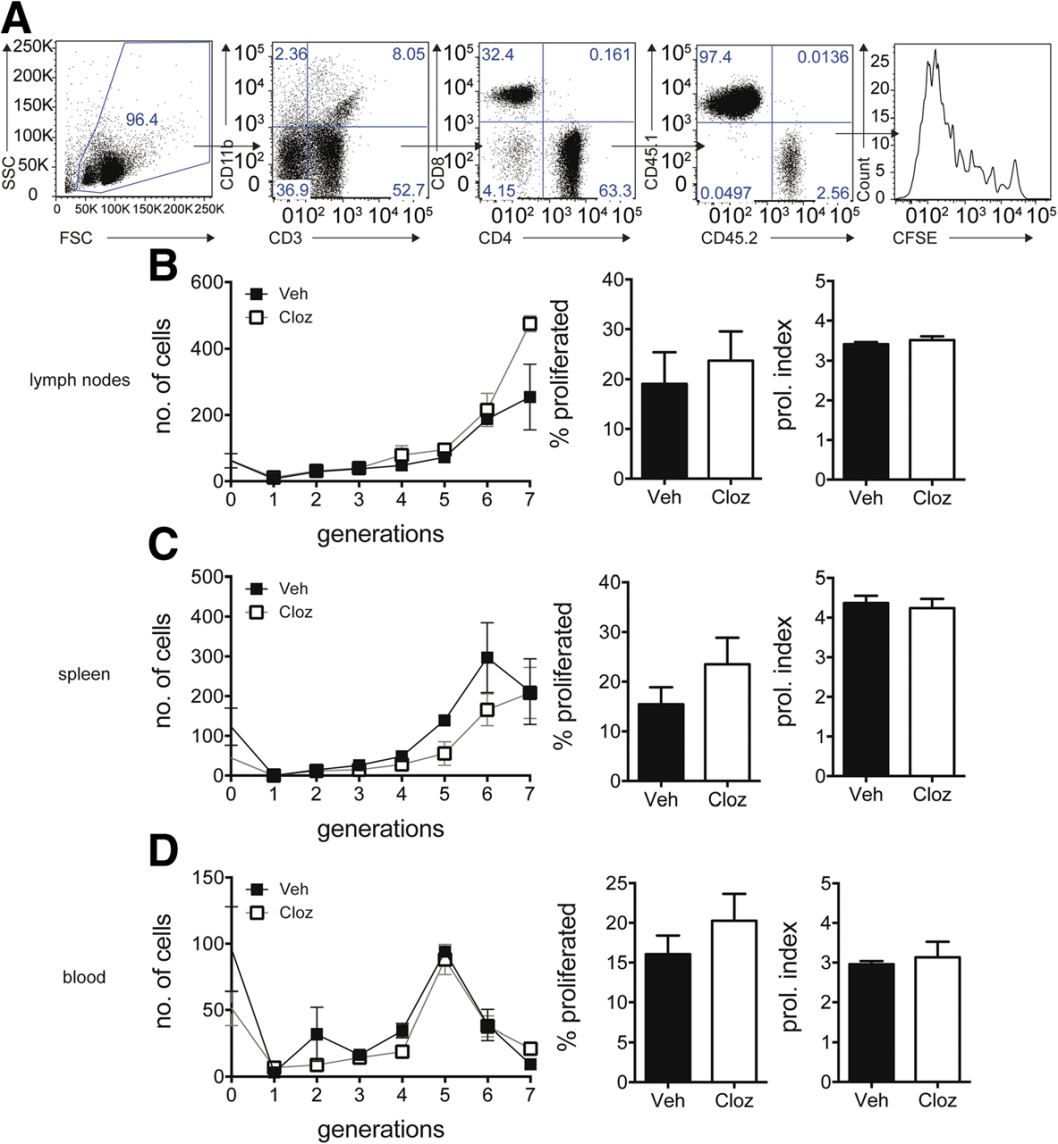
Clozapine did not alter Th cell subsets in the periphery. **a–d** In vivo proliferation. Gating strategy to identify adoptively transferred 2D2 MOG_35-55_ TCR CD4 cells (**a**). Proliferation assessed by % proliferated and proliferation index in **b** blood, **c** spleen, and **d** draining lymph nodes. *p* > 0.05 by Student’s *t* test. Shown is one representative experiment of two (*n* = 3 mice in each group)

CNS inflammation and demyelination in MS and EAE is dependent on the function of CD4^+^ T cells [15]. EAE is induced by immunization with a myelin self-peptide such as MOG_35-55_, which causes a rapid expansion of antigen-specific CD4^+^ T cells that initiate inflammation in the CNS [15]. Given the requirement for CD4 T cell expansion, defects in T cell proliferation can reduce the severity of EAE [16, 17], and in contrast, failure to suppress proliferation exacerbates EAE as seen in IFN-γ [18] or Treg-deficient mice [17]. A previous study reported that the atypical antipsychotic agent, quetiapine, reduced disease in EAE by impairing CD4 T cell proliferation [11]. Given that CD4 T cells express dopaminergic receptors (data shown in Additional file 1) as well as other neurotransmitter receptors targeted by atypical antipsychotic agents [19, 20], we investigated whether clozapine had affected MOG_35-55_-specific T cell expansion. To address this, we used an established in vivo model of proliferation [16]. We show that clozapine-treated mice maintained robust CD4^+^ T cell expansion as assessed by the percentage of cells that proliferated and the proliferative index in the peripheral blood (Fig. 2b), spleen (Fig. 2c), and draining lymph nodes (Fig. 2d) indicating that orally administered clozapine does not affect MOG-specific CD4^+^ expansion in vivo.

### Clozapine did not alter pathogenic T cell subsets but promoted Tregs

EAE is a disease that is driven by pathogenic Th1 cells expressing T-bet and IFN-γ as well as Th17 cells expressing RORγT and IL-17A. This dependence was demonstrated when it was shown that mice which have defective Th1 and Th17 development (i.e., IL-12p40-deficient mice) were resistant to EAE [21]. In addition, severity of EAE is regulated by Foxp3-expressing regulatory T cells (Tregs) that have immunosuppressive capacity [22]. Clozapine has previously been shown to decrease production of IFN-γ and enhance IL-4 and IL-10 in poly (I:C)-stimulated PBMC cultures from schizophrenia patients, which suggests an effect on CD4^+^ T cell differentiation [23]. To assess development of Th1, Th17, and Treg cell subsets during EAE, we measured RORγT, T-bet, and Foxp3 expression in peripheral CD4^+^ T cells during EAE as these transcription factors are master regulators of Th1 [24], Th17 [25], and Treg [26] cells, respectively.

**Fig. 3:**
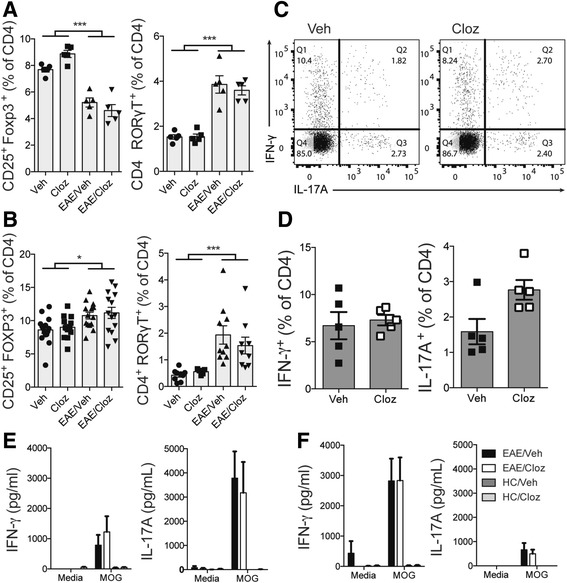
Clozapine did not alter Th cell subsets in the periphery. Tregs and Th17 cells were analyzed by flow cytometry 5 d.p.i (**a**) and 15 d.p.i (**b**; gating strategy shown in Additional file 3). Shown are the means and SEM of individual mice from one experiment with 5/group (**a**) or 2–3 experiments combined with 10–15 mice/group (**b**). **c** Representative dot plots of intracellular cytokine staining from spleen 15 d.p.i. **d** Graph of IFN-γ^+^ and IL-17A^+^ cells presented as percentage of CD4. Shown are means and SEM of individual mice from one experiment (*n* = 5 mice/group). **e** Splenocytes were re-stimulated with MOG peptide at 5 d.p.i for 72 h and IFN-γ and IL-17A in the supernatant measured by ELISA. Shown are means and SEM of individual mice from one experiment. Shown are the means and SEM of individual mice from one experiment (*n* = 5 mice/group). **f** Splenocytes were re-stimulated with MOG peptide 15 d.p.i for 72 h. IFN-γ and IL-17A in the supernatant measured by ELISA. Shown are the means and SEM of individual mice from an experiment representative of four (*n* = 5 mice/group). Statistical analyses were performed using two-way ANOVA and Sidak’s multiple comparison test

At 5 d.p.i, mice with EAE had a lower proportion of Tregs (CD25^+^ FoxP3^+^) in the spleen than unimmunized mice while mice with EAE had an increased proportion of Th17 (RORγT^+^) when compared to unimmunized mice. No difference in Tregs or Th17 cells was measured between vehicle and clozapine-treated mice independent of EAE (Fig. 3a). In contrast to the induction phase of EAE, in the effector phase (i.e., 15 d.p.i), we observed a subtle but significant increase in Tregs while the significant increase in Th17 cells induced by EAE persisted. Clozapine treatment had no effect on Tregs or Th17 cells in immunized or unimmunized mice (Fig. 3b). T-bet expression was not detected by flow cytometry, and instead, we used intracellular cytokine staining for IFN-γ and IL-17A to identify Th1 and Th17 cells, respectively (Fig. 3c), and found that IFN-γ^+^ and IL-17A^+^ CD4 T cells were unaffected by clozapine treatment (Fig. 3d). These results were verified when no difference in MOG_35-55_-specific IFN-γ and IL-17A recall responses was detected between vehicle and clozapine-treated animals at 5 d.p.i (Fig. 3e) and 15 d.p.i (Fig. 3f). It is worth noting that we have previously found IL-17A in the supernatant to be decreased after MOG_35-55_-specific recall response in risperidone-treated mice [10], suggesting that clozapine may have a distinct mechanism of action to that of risperidone.

**Fig. 4:**
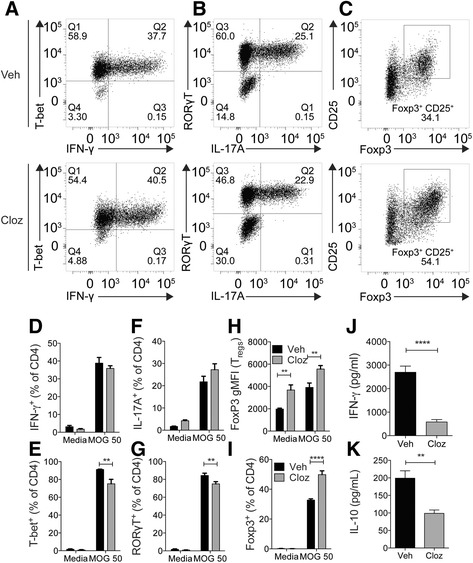
Clozapine promoted Treg expansion in an in vitro model of T cell differentiation. 2D2 TCR^MOG35-55^ splenocytes were cultured at 1 × 10^6^ cells/well in Th differentiation medium and stimulated with MOG_35-55_ peptide for 72 h. Representative dot plots of (**a**) T-bet and IFN-γ in Th1 differentiating medium, **b** RORγT and IL-17A in Th17 differentiating medium, and **c** CD25 and Foxp3 in Treg differentiating medium gated on CD4^+^ T cells after 72 h of stimulation with MOG_35-55_ with clozapine (20 μM) or vehicle. Graphical presentation of IFN-γ^+^ (**d**), IL-17A^+^ (**f**), or CD25^+^ Foxp3^+^ (**h**) T cells. Graphical representation of T-bet^+^ (**e**) and RORγT^+^ (**g**) as percentage of CD4. Foxp3 expression presented as geometric MFI in all CD4 T cells after differentiating to Tregs (**i**). IFN-γ (**j**) and IL-10 (**k**) in the supernatant from splenocytes stimulated with MOG_35-55_ peptide for 72 h. ****p < 0.0001 as assessed by one-way ANOVA with Dunnett’s multiple comparison test. **p < 0.01, ****p < 0.0001 by Mann-Whitney test. Shown is a representative experiment of 4

To understand the effect of clozapine on the differentiation of T cells, we stimulated splenocytes from 2D2 TCR^MOG35-55^ mice with MOG_35-55_ peptide under conditions to induce Th1 (Fig. 4a), Th17 (Fig. 4b), and induced Tregs (iTregs) (Fig. 4c). When we added clozapine into the culture medium, we did not detect a significant difference in the induction of Th1 or Th17 cells measured by IFN-γ^+^ (Fig. 4d) or IL-17A^+^ (Fig. 4e) CD4 T cells. We did however measure a subtle but statistically significant decrease in T-bet^+^ (Fig. 4f) and RORγT^+^ (Fig. 4g) T cells in the presence of clozapine. Interestingly, we measured a significant increase in Foxp3 protein expression in iTregs (Fig. 4h) indicating that clozapine promotes Foxp3 expression. Additionally, the proportion of iTregs significantly increased from 32.7% of CD4^+^ to 49.8% (Fig. 4i) in the presence of clozapine; altogether, these results indicate that clozapine promotes the generation of iTregs and Foxp3 expression. Despite minimal difference in Th1 or Th17 cell differentiation, we measured significantly less IFN-γ (Fig. 4j) and IL-10 (Fig. 4k) in the supernatant after stimulating with MOG_35-55_ peptide alone, suggesting a possible alteration in downstream effector functions.

### Protection from EAE by clozapine was not dependent on regulatory CD4^+^ T cells

**Fig. 5:**
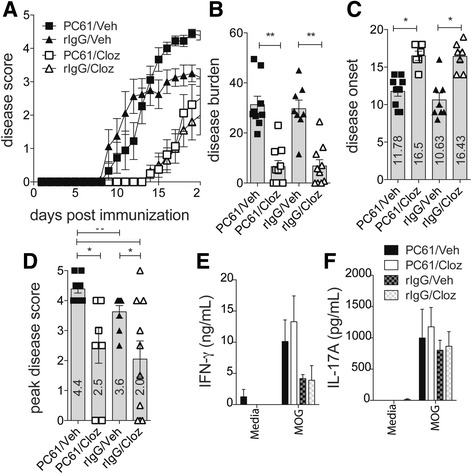
Clozapine was protective independent of regulatory T cells. **a–f** Tregs were neutralized in mice 3 days before EAE induction and maintained throughout the experiment. Clozapine was added to drinking water 1 day before induction for EAE and mice scored daily for disease (0: normal to 5: moribund). Disease score of mice (**a**). Disease burden assessed by area under the curve analysis (**b**). Onset of clinical disease (**c**). Peak disease score reached of mice with disease (**d**). Shown are the means and SEM of individual mice from two experiments combined (**a–d** *n* = 5 mice/group). *p < 0.05, **p < 0.01 by two-way ANOVA and Sidak’s multiple comparison test. ••*p* < 0.01 by Mann-Whitney test. **e–f** IFN-γ (**e**) and IL-17A (**f**) in the supernatant after MOG_35-55_-peptide-specific recall for 72 h. Shown are means and SEM of individual mice from one experiment (*n* = 5 mice in each group)

Our results indicate that in vitro, clozapine enhances the development of CD25^+^ Foxp3^+^ Tregs. Tregs are known to limit inflammatory damage during EAE, and their importance was highlighted by the depletion of CD25^+^ or Foxp3^+^ T cells during EAE, which resulted in exacerbated disease [27, 28]. Since Tregs are protective in this model, we questioned whether promotion of Treg differentiation by clozapine was responsible for its protective effect during EAE. We neutralized Treg cells in vivo prior to EAE induction by administration of anti-CD25 antibody. This treatment was maintained throughout the experiment and effectively blocked CD25 (data shown in Additional file 2). We found that neutralization of Tregs exacerbated disease in untreated mice (Fig. 5a) whereas clozapine-treated mice remained protected from disease and sustained a lower overall disease burden (Fig. 5b) and maintained a delayed onset of disease (Fig. 5c) and peak disease score (Fig. 5d) paralleling mice which did not receive anti-CD25. This data demonstrates that Treg function is dispensable for protection by clozapine.

### Clozapine did not alter encephalitogenicity of MOG_35-55_-specific CD4^+^ T cells

**Fig. 6:**
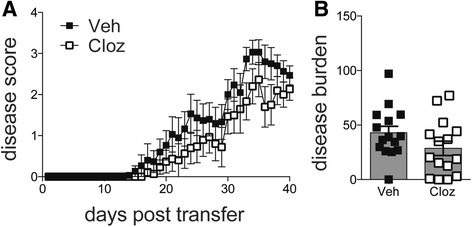
Clozapine did not alter encephalitogenic capacity of T cells to induce EAE. Passive EAE was induced in mice by injection of MOG-specific cells expanded either in vehicle or clozapine. Disease score of mice (**a**). Disease burden of mice assessed by area under the curve (**b**). Shown are the means and SEM of individual mice from three experiments combined (*n* = 15 mice/group). Mann-Whitney test was used to determine whether disease burden (i.e., AUC) was statistically different between groups

Our in vitro model of T cell differentiation indicates that clozapine does not alter the differentiation of CD4 T cells into Th1 and Th17 cell subsets. However, these results show some potential for clozapine to alter T cell effector functions given that we measured less IFN-γ and IL-10 in the supernatant when stimulated with MOG. While our study has shown that Tregs do not mediate protection in clozapine-treated mice, it is possible that clozapine could be affecting the encephalitogenic capacity of CD4^+^ T cells. We induced passive EAE by injecting MOG_35-55_-specific T cells expanded in vitro in the presence of clozapine into naïve mice. Adoptive transfer of encephalitogenic T cells induced EAE with a similar disease progression (Fig. 6a) and disease burden (Fig. 6b) independent of whether clozapine was present during in vitro expansion indicating that clozapine does not affect the encephalitogenic capacity of CD4 T cells.

## Discussion

Atypical antipsychotics like clozapine readily cross the BBB and are used for treating psychiatric diseases like schizophrenia. Recently, these medicines have been recognized as immune-modulating agents as they are able to suppress inflammation associated with schizophrenia [29]. Few studies are available describing the effects of clozapine treatment on the immune response, but these have shown that clozapine can inhibit the activation of immune cells like microglia and T cells in response to inflammatory stimuli [12, 30, 31]. Given that both schizophrenia and multiple sclerosis are inflammatory diseases of the central nervous system and that clozapine is able to reduce inflammation in the CNS, we wished to investigate whether clozapine could be re-purposed to treat progressive forms of multiple sclerosis for which there is only one effective therapeutic option available [5]. Indeed, we have shown that clozapine is able to reduce disease in EAE, indicating that it is a great candidate for multiple sclerosis; however, the precise mechanism is not yet known. In this study, we show that clozapine treatment does not significantly alter the development of robust antigen-specific T cell responses despite effective suppression of EAE disease.

The effect of clozapine on T cell activation has previously been investigated in human PBMCs and is consistent with our findings, showing that clozapine inhibits secretion of IFN-γ after stimulation of CD3 and CD28 [31]. In our study, we showed that clozapine inhibits IFN-γ secretion in response to an antigen; however, in contrast to the previous study, we did not observe significant alterations in T cell differentiation to Th1 or Th17 in vitro or during EAE.

One of the most striking findings in this study is that clozapine promotes in vitro differentiation of Tregs and expression of Foxp3, a key transcription factor for the development and function of Tregs. Although clozapine had the potential to augment Treg function, it did not appear to be important for disease protection during EAE as neutralization of CD25^+^ Tregs had no effect on disease protection. Interestingly, while clozapine promoted Treg differentiation, little effect was observed during differentiation to Th1 and Th17 cells. This observation could be explained by clozapine activating the Akt pathway as has reported in various tissues [32–34], and since this pathway is important for CD3, CD28 and cytokine signalling [35]. Akt is upstream of the mammalian target of rapamycin (mTOR) and regulates T cell differentiation during activation. For example, suppression of mTOR activity promotes the generation of Foxp3^+^ Tregs [36], and the deletion of mTOR in CD4 T cells enhances differentiation of Tregs but not Th1, Th2, or Th17 cells [37], indicating a potential role for mTOR in the mechanism of action of clozapine.

Despite the effects of clozapine in an in vitro differentiation model, we did not find that these alterations were important for disease protection during EAE as no difference in Th1, Th17, or Treg differentiation was detected with treatment during the induction and effector phase of disease even though mice were protected from disease. This suggests that protection may not be mediated through alteration of the T cell response directly. It is possible that clozapine alters the ability of macrophages and microglia to become activated and initiate disease in the CNS independent of CD4 T cells, although this is yet to be shown. However, clozapine has recently been shown to alter the production of inflammatory cytokines from in vitro cultured microglia [30], and we have shown previously that clozapine suppresses the production of IL-12 in bone marrow-derived macrophages stimulated with lipopolysaccharide indicating that clozapine is able to alter macrophage activation [10]. This effect may be important given that once autoreactive T cells initiate the inflammatory response in the CNS, inflammatory monocytes are recruited and their numbers correlate to severity of disease [38]. The ability of clozapine to alter macrophage activation to be less inflammatory may contribute to attenuated disease during treatment as these cells are predominant in the demyelinating regions of the brain in EAE and MS [39].

In conclusion, while clozapine is effective at reducing EAE in mice, we did not find defective antigen-specific T cell responses. Given that we have recently shown clozapine to inhibit the activation of microglia in the CNS during EAE [14] and that clozapine alters the activation of LPS-stimulated macrophages directly [10], instead, we believe the mechanism of protection may involve other immune pathways including the alteration of myeloid activation in the CNS specifically. We have recently shown that clozapine effectively reduces established EAE disease when given therapeutically at 12 or 20 d.p.i, indicating that clozapine has potential as a therapy for MS [14]. Finally, it has been suggested that prospective therapies for progressive MS have immune-modulating properties that target myeloid cell activation in the CNS and readily cross an intact BBB [40], further supporting the feasibility of using clozapine as a potential therapy for progressive MS.

## Additional files

Additional file 1: Gating strategy for identifying dopaminergic and adrenergic receptors on T cells. Spleens were isolated from mice 15 d.p.i and analyzed by flow cytometry for dopamine and adrenergic receptor expression. A gating strategy for identifying CD4 (bottom left), CD8 (bottom center), and Tregs (bottom right). Representative histograms for expression of B dopamine D1 receptor (D1R) and C dopamine D2 receptor (D2R) on CD4 T cells. Graphical representation of receptor expression on different T cell subsets of D D1R and E D2R. Shown are means and SEM of individual mice from one experiment (*n* = 5 mice in each group). Statistical analyses between healthy control and EAE mice conducted by two-way ANOVA. (TIF 17402 kb)

Additional file 2: CD25 neutralization 0, 10, and 17 d.p.i Mice were injected with 200 μg/mouse anti-CD25 (PC61) antibody at −3, 7, and 14 d.p.i CD25 neutralization was assessed by flow cytometry using Alexa Fluor 488 conjugated anti CD25 (PC61) at days A 0, B 10, and C 17. CD4+ T cells were identified using the gating strategy described in Additional file 3. (TIF 12283 kb)

Additional file 3:Gating strategy to identify CD4+ T cells and 2D2 MOG35-55 TCR CD4+. Single cells were gated using FSC-A and FSC-H. Cells of interest were gated using FSC-A and SSC-A. Lymphocytes were identified by CD11b− and CD3+ expression. CD4 T cells were distinguished using CD8− and CD4+ expression. Tregs were identified as CD25+ and Foxp3+. Th17 cells were identified as ROR$\gamma$T+. (TIF 14847 kb)

## Abbreviations

BBB: Blood-brain barrier
CNS: Central nervous system
d.p.i: Days post immunization
EAE: Experimental autoimmune encephalomyelitis
i.p: Intraperitoneal
IL: Interleukin
iTreg: Induced Treg
MOG: Myelin oligodendrocyte glycoprotein
MS: Multiple sclerosis
PMA: Phorbol 12-myristate 13-acetate
s.c: Subcutaneous
TCR: T cell receptor
TGF: Transforming growth factor
Treg: Regulatory T cell
