# Supplementary figures and images for: Amelioration of experimental autoimmune encephalomyelitis by clozapine is not associated with defective CD4 T cell responses
Source: J Neuroinflammation. 2017 Mar 29;14:68. doi: 10.1186/s12974-017-0842-5 (PMC5372297; doi:10.1186/s12974-017-0842-5)

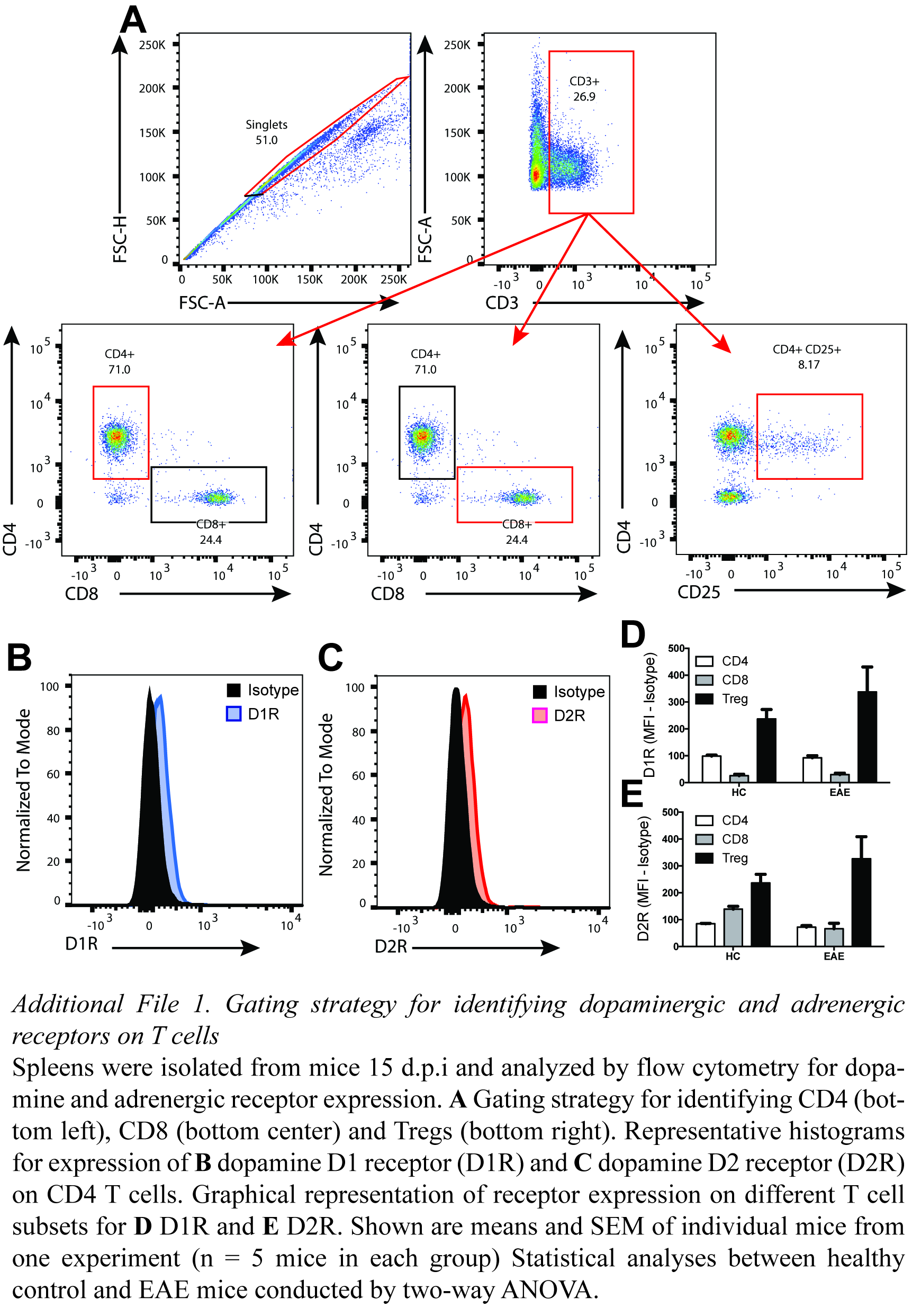

Supplement: Supplementary file 1 — Gating strategy for identifying dopaminergic and adrenergic receptors on T cells. Spleens were isolated from mice 15 d.p.i and analyzed by flow cytometry for dopamine and adrenergic receptor expression. A gating strategy for identifying CD4 (bottom left), CD8 (bottom center), and Tregs (bottom right). Representative histograms for expression of B dopamine D1 receptor (D1R) and C dopamine D2 receptor (D2R) on CD4 T cells. Graphical representation of receptor expression on different T cell subsets of D D1R and E D2R. Shown are means and SEM of individual mice from one experiment (n = 5 mice in each group). Statistical analyses between healthy control and EAE mice conducted by two-way ANOVA. (TIF 17402 kb) [file 12974_2017_842_MOESM1_ESM.tif]

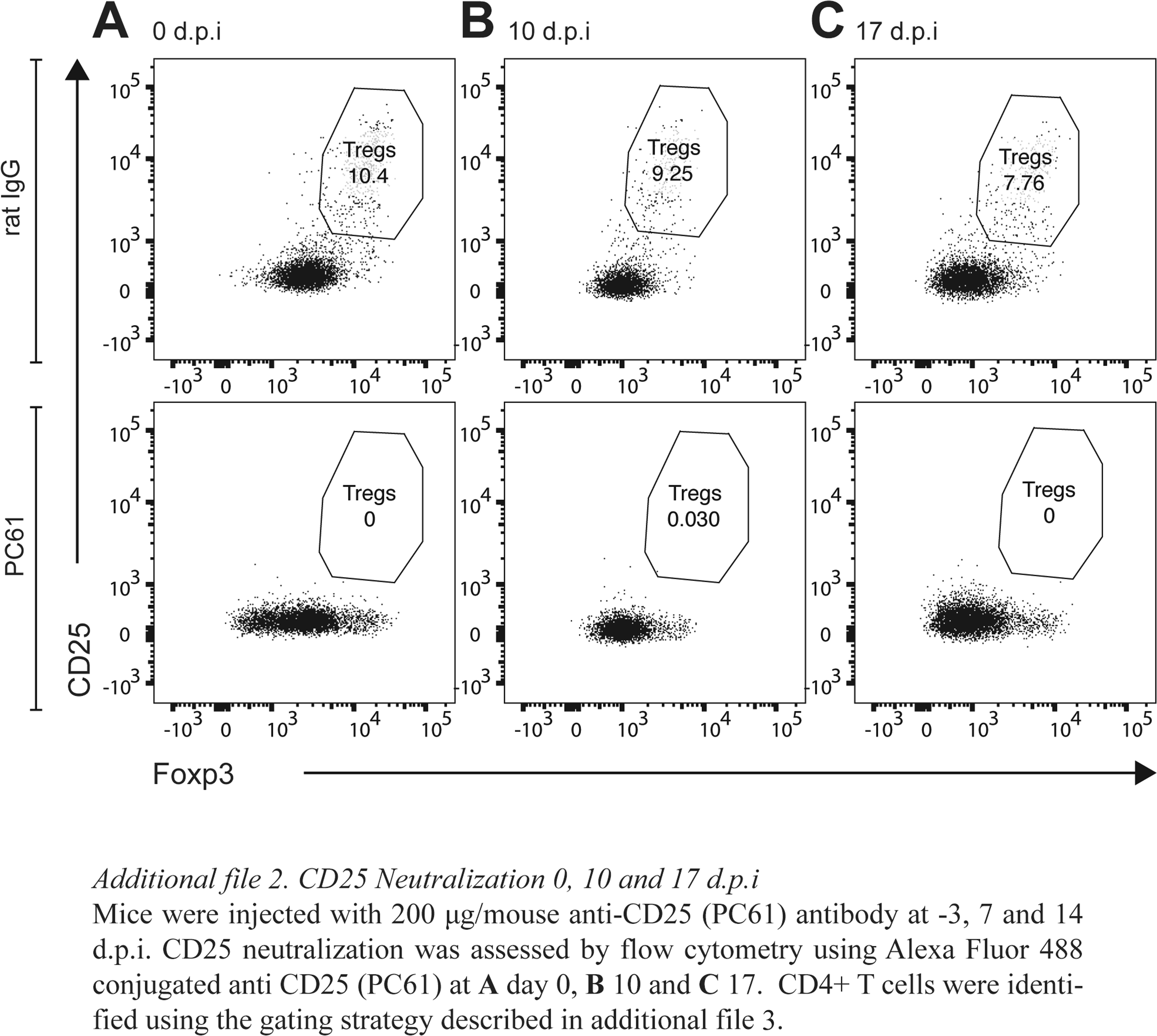

Supplement: Supplementary file 2 — CD25 neutralization 0, 10, and 17 d.p.i Mice were injected with 200 μg/mouse anti-CD25 (PC61) antibody at −3, 7, and 14 d.p.i CD25 neutralization was assessed by flow cytometry using Alexa Fluor 488 conjugated anti CD25 (PC61) at days A 0, B 10, and C 17. CD4+ T cells were identified using the gating strategy described in Additional file 3. (TIF 12283 kb) [file 12974_2017_842_MOESM2_ESM.tif]

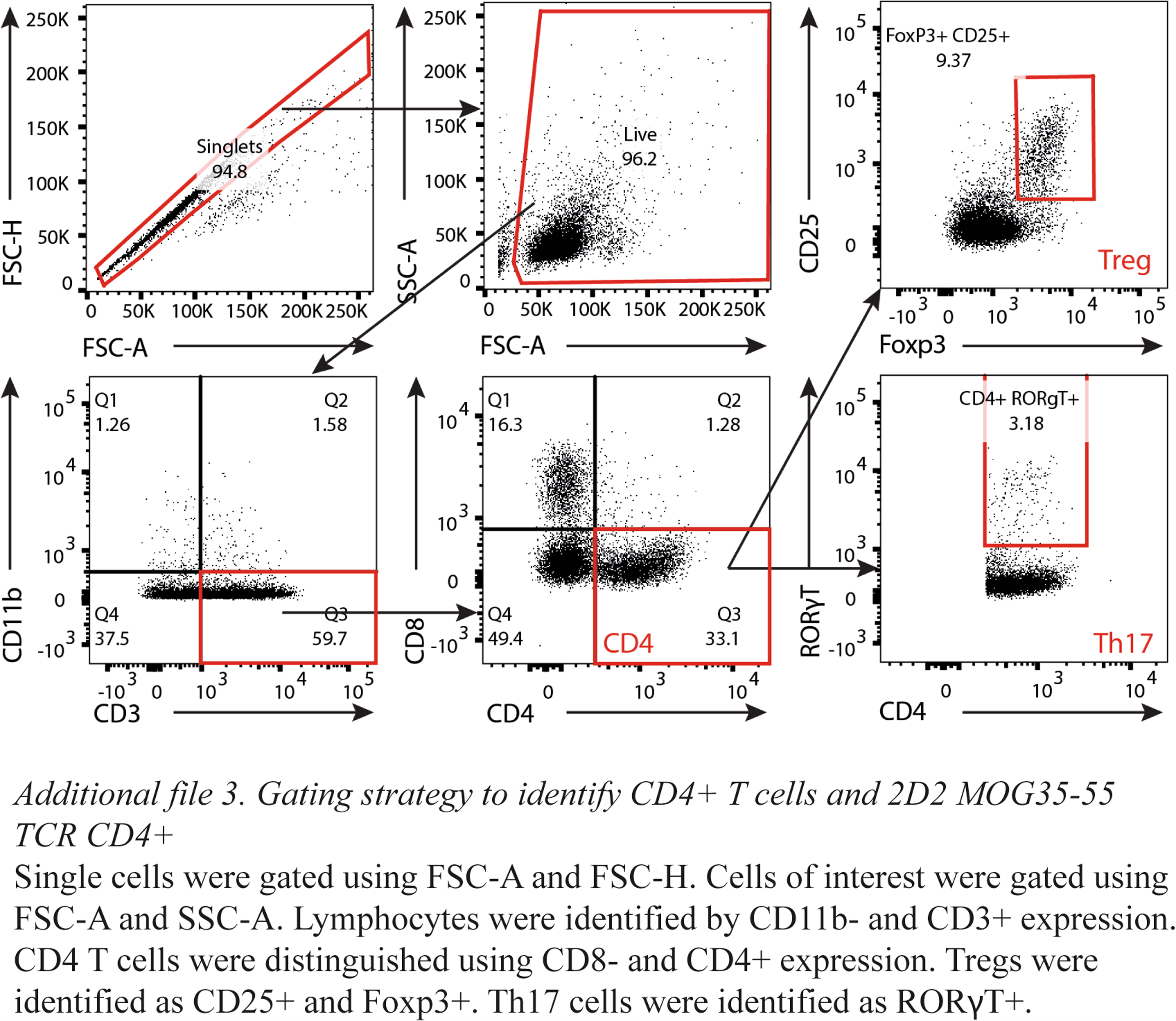

Supplement: Supplementary file 3 — Gating strategy to identify CD4+ T cells and 2D2 MOG35-55 TCR CD4+. Single cells were gated using FSC-A and FSC-H. Cells of interest were gated using FSC-A and SSC-A. Lymphocytes were identified by CD11b− and CD3+ expression. CD4 T cells were distinguished using CD8− and CD4+ expression. Tregs were identified as CD25+ and Foxp3+. Th17 cells were identified as ROR$\gamma$γT+. (TIF 14847 kb) [file 12974_2017_842_MOESM3_ESM.tif]
